# A 19-year-old man with sickle cell disease presenting with spinal infarction: a case report

**DOI:** 10.1186/1752-1947-7-210

**Published:** 2013-08-23

**Authors:** April Edwards, E Leila Jerome Clay, Valerie Jewells, Stacie Adams, Regina D Crawford, Rupa Redding-Lallinger

**Affiliations:** 1 Departments of Internal Medicine and Pediatrics, University of North Carolina School of Medicine, 101 Manning Drive, Chapel Hill, NC 27514, USA; 2 Departments of Pediatrics and Internal Medicine, Division of Hematology and Oncology, University of North Carolina School of Medicine, 170 Manning Drive 1185A, Physician Office Building CB#7236, Chapel Hill, NC 27599-7236, USA; 3 Departments of Pediatrics and Internal Medicine, Division of Hematology and Oncology, Georgia Regents University, 1120 15th Street, BH 2015, Augusta, GA 30912, USA; 4 Department of Radiology, University of North Carolina School of Medicine, 100 Manning Drive, Radiology CB#7510, Old Clinic Building, Chapel Hill, NC 27599-7510, USA; 5 Department of Pediatrics, Michigan State University, GRMEP 1000 Monroe Avenue, NW, Grand Rapids, MI 49503, USA; 6 Department of Medicine, Division of Hematology, Duke University Medical Center, 2212 Elba Street DUMC Box 3939, Durham, NC 27705, USA

## Abstract

**Introduction:**

Vasculopathy of the large vessels commonly occurs in sickle cell disease, and as a result cerebral infarction is a well characterized complication of this condition. However, spinal infarction appears to be rare. Spinal infarct is infrequent in the non-sickle cell population as well, and accounts for only about 1 percent of all central nervous system infarcts.

**Case presentation:**

In the present work, we report the case of a 19-year-old African-American man with sickle cell disease who experienced an anterior spinal infarct and subsequent quadriplegia. He was incidentally noted to be a heterozygote for factor V Leiden. We also reviewed the literature and found two previous cases of spinal cord infarction and sickle hemoglobin. Our literature search did not demonstrate that heterozygocity for factor V Leiden plays an important role in spinal cord infarction.

**Conclusions:**

The paucity of cases associated with sickle hemoglobin does not allow us to postulate any particular risk factors with sickle cell disease that might predispose patients to spinal cord infarction. Our patient’s case raises the question as to whether spinal cord infarction is being missed in individuals with sickle cell disease and neurologic symptoms.

## Introduction

Cerebral infarction is the most common neurologic complication that occurs with sickle cell disease (SCD); it can be either overt or silent and it can be associated with significant morbidity [1]. Overt stroke in SCD was first characterized in 1923, and histopathologic studies later revealed large vessel narrowing with superimposed thrombosis as the underlying cause [2,3]. Though cerebral infarction is the most frequent neurological complication, a number of other potentially devastating central nervous system (CNS) sequelae have also been described. These include: intra-cranial hemorrhage, isolated neuropathies, transverse myelitis, auditory and ocular manifestations, and spinal cord involvement [1]. In the spinal cord there has been a description of cord compression by extramedullary hemopoietic tissue in addition to rare case reports of spinal cord infarction [1,4-6].

In the non-sickle cell disease population it appears that spinal infarct is much less frequent than cerebral infarction as well, and accounts for only about 1 percent of all CNS infarcts [7]. Of those with spinal infarction, most appear to be from traumatic or surgical etiologies than other organic causes [7,8]. Aortic disease is a frequent culprit with many case reports detailing adverse sequelae following surgical repair of aneurysms, but also aortic thrombosis, and aortic dissection [8]. Other non-traumatic, non-surgical etiologies of spinal cord infarct include: global hypotension and/or arterial insufficiency, often after cardiac arrest; transient ischemic attacks; fibrocartilagenous emboli; arterial vascular malformations; syphilitic arteritis and adjacent spinal disease [8-10]. In a 2006 study, Novy *et al*. noted that 12 of their 27 patients with spinal infarct had pre-existing spinal disease including compression fractures, spondylolisthesis, chronic arachonoiditis and chronic cervical disk protrusion, and of those 12 patients, 11 had an infarct at the level of their pre-existing disease [7]. However, the cause of spinal infarct is frequently cryptogenic [11].

There is considerable evidence that sickle cell disease represents a hypercoagulable state [12-15]. It appears that nearly every component of hemostasis is altered to some degree in SCD [15]. Studies have indicated that in sickle cell disease there is increased platelet activation and aggregation, increased levels of D-dimer and fibrinogen and fibrin-fibrinogen complex while there are simultaneously decreased factors V, VII, VIIa and proteins C and S [13]. There is good evidence that there is externalization of phosphatidylserine (a phospholipid normally found in the inner monolayer of red blood cells) in SCD, which is thought to play a significant role in promoting macrophage recognition in erythrophagocytosis and thus triggering a signal for the coagulation process [13,14]. Increased phosphatidylserine exposure is also thought to be associated with increased tissue factor expression [14]. However, it remains unclear how or to what extent those abnormalities contribute to disease complications such as cerebral and spinal infarcts.

Because cerebral infarcts occur only in a subset of the sickle cell population, it has been postulated that there may be identifiable features of this subgroup that exacerbate the hypercoagulable state of sickle cell disease. In the search for possible characteristics of this subpopulation, some have begun to explore factors that predispose the general population to coagulation abnormalities and thrombophilia. Specifically, there have been case reports of persons with SCD who developed CNS infarcts and were found to have the factor V Leiden, a prothrombin gene variant, a methylenetetrahidrofolate reductase gene mutation, or some combination of those mutations [16-18]. These studies were conducted in Brazil and Israel; notably the prevalence of the factor V Leiden and the prothrombin gene variant are known to be very low in African-Americans [19]. Also, there have been a few single nucleotide polymorphisms (SNPs) in persons with SCD that have been found to be associated with increased stroke risk: ANXA2, TGFBR3, and TEK were noted in a study including these SNPs [20]. However, further validation is needed before these can be used to prospectively guide recommendations for molecular genetic testing or treatment [20]. There is no known identifiable thrombophilic abnormality that predicts cerebral infarction in sickle cell disease.

## Case presentation

On the morning of admission, our patient, a 19-year-old African-American man with sickle cell anemia, felt himself to be in his usual state of health, although he had just been discharged the previous day from a hospitalization for acute chest syndrome. He ate breakfast and spent the day watching television. However, at approximately 5:45 p.m. when he used the bathroom, he noticed that he could not pull up his trousers due to weakness in his left arm. As he walked out of the bathroom, he noted that he was having difficulty walking because of weakness in his right leg. As his mother was helping him to his bed, his left leg also became weak. He began experiencing ‘shocking’ pains on both sides of his neck, which were unlike his usual pain, and also noted that he had an erection. These events transpired rapidly, within about six minutes, at which point his family called Emergency Medical Services (EMS) and our patient was transported to our hospital.

On arrival at our hospital, he was alert and oriented and cranial nerves II to XII were intact. He had flaccid paralysis of the bilateral upper extremities and the left lower extremity, and normal tone with 5 out of 5 strength in the right lower extremity. He had areflexia in the biceps, triceps, and brachioradialis bilaterally, hyper-reflexia at the left patella, and sustained clonus at the left Achilles. Sensation was intact throughout. The results of the rest of his physical examination were normal.

Relevant medical history included asthma, recurrent acute chest syndrome (>10 episodes), and intermittent attempts at hydroxyurea treatment with poor compliance over the previous 10 years. Following the identification of silent cerebral infarcts, he was treated for the three years between 2005 and 2008 with exchange transfusions to maintain hemoglobin S < 30 percent; during this time he did very well. At 10 days prior to presentation, he was hospitalized with an acute chest syndrome. During that hospitalization he had an initial PO_2_ of 76, a hemoglobin (Hb)/hematocrit (Hct) nadir of 5.8/17, and was found to have a methicillin-resistant *Staphylococcus aureus* (MRSA) pneumonia. He was treated with antibiotics and a transfusion. His discharge hemoglobin was 6.6 and oxygen saturation 96 percent. He was without symptoms at the time of discharge.

**Figure 1:**
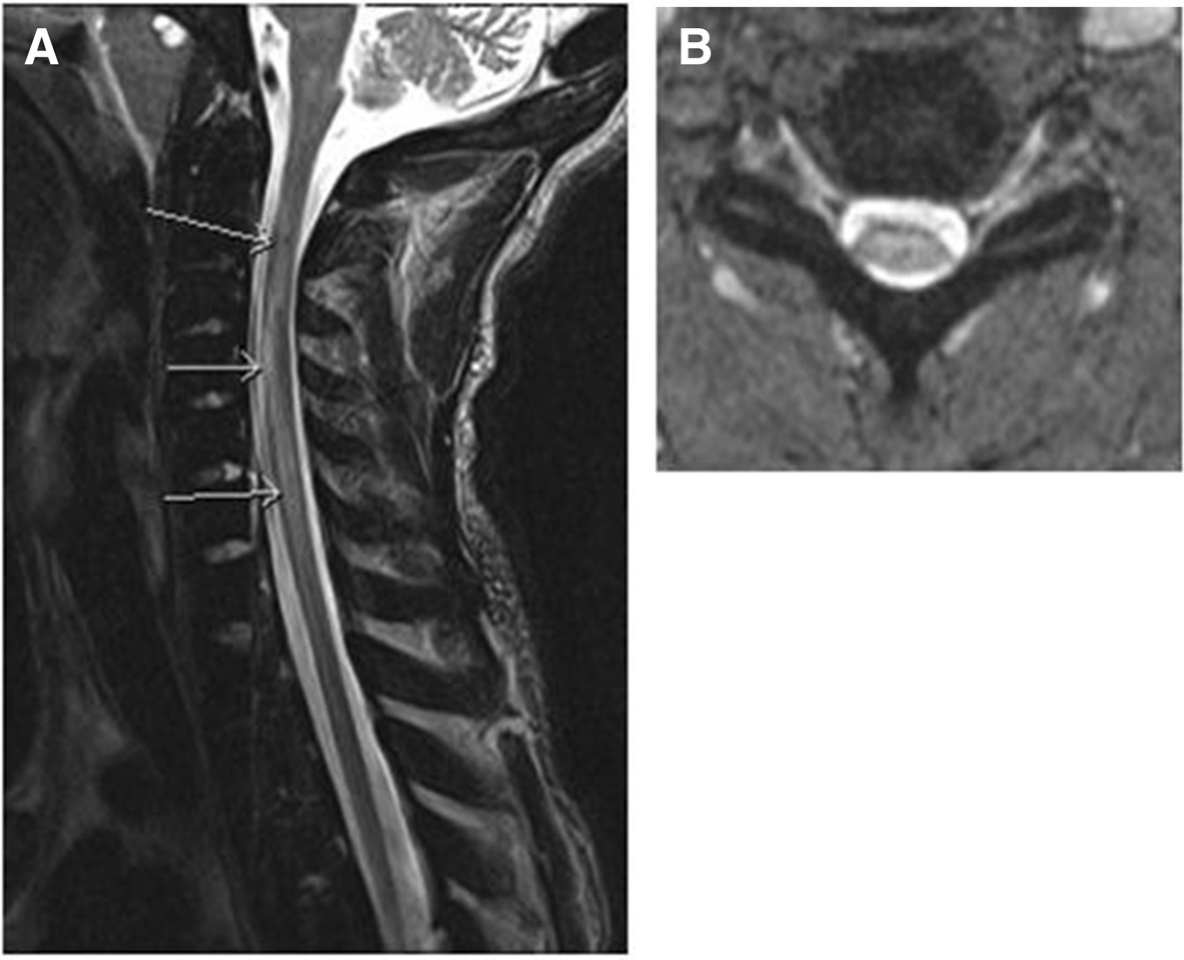
**A,B T2 hyperintense signal extending from C2 to C7 with edema of the gray and white matter of the cord.** The arrows point to the edema. As with all infarcts, the area of infarct is bright on B1000 and dark on apparent diffusion coefficient sequences.

Admission laboratory test data included a white blood cell count of 12 × 10^3^/uL Hb 8.7g/dL, Hct 26 percent, platelets 449 × 10^3^ cells/mm^3^ with a hemoglobin electrophoresis of HbA 86 percent, HbS 7 percent, and HbC 7 percent. He had a lumbar puncture that demonstrated unremarkable cerebrospinal fluid findings and no evidence of IgG oligoclonal bands. The results of peripheral blood and urine cultures were negative. A chest X-ray showed patchy consolidation in the right upper lobe suspicious for pneumonia. The results of computed tomography (CT) angiography of the head and neck were unremarkable. Given concern for spinal cord involvement, 1.5T T1, T2, and fluid attenuated inversion recovery (FLAIR) magnetic resonance imaging (MRI) studies of the brain and cervical spine was performed showing an abnormal T2/FLAIR signal in the cervical spinal cord, which was thought at that time likely to be due to artifact. Later the initial MRI was read to also show swelling of the cord in the same area. He was admitted to the neurologic intensive care unit where he received an exchange transfusion with no significant improvement in his symptoms; subsequent hemoglobin electrophoresis showed HbA 85 percent, HbS 9 percent. While in the intensive care unit (ICU) he experienced episodes of hypotension that were initially managed with vasopressors. After his blood pressure stabilized he was transitioned to fludrocortisone and midodrine. He never had respiratory insufficiency. Two days after admission he had a repeat MRI, which showed T2 hyperintense signal extending from C2 through to C7 (Figure 1A). In addition, diffusion-weighted imaging demonstrated restricted diffusion consistent with a focus of infarction in addition to cord edema and swelling in the gray and white matter of the right side of the cord. There was associated enlargement of the spinal cord consistent with edema from the anterior spinal infarct. A hypercoagulability investigation performed during his hospitalization included a polymerase chain reaction (PCR) study that demonstrated that he was heterozygous for the factor V Leiden 1691 G>A mutation. Other studies performed were for factor VIII, fibrinogen, functional anti-thrombin, lupus anti-coagulant, anti-cardiolipin, all of which were within normal limits. His erythrocyte sedimentation rate (ESR) and C-reactive protein (CRP) levels were both elevated, and proteins C and S were found to be low but within the expected range for someone with sickle cell disease. He was anti-coagulated with a heparin drip during his stay in the acute care facility, but this was discontinued on discharge. A monthly exchange transfusion regimen was instituted with the goal of keeping his hemoglobin S level < 30 percent.

Although initially there was almost complete paralysis of his extremities, over the four days he spent in the neurologic ICU, our patient demonstrated slow but steady progress in regaining some motor function of his affected limbs. He was transferred from the ICU to the wards on day five and began working with physical and occupational therapy. On day 10, he was transferred to a rehabilitation facility, where he made gradual but steady progress in regaining motor function. He was discharged home after three weeks.

**Figure 2:**
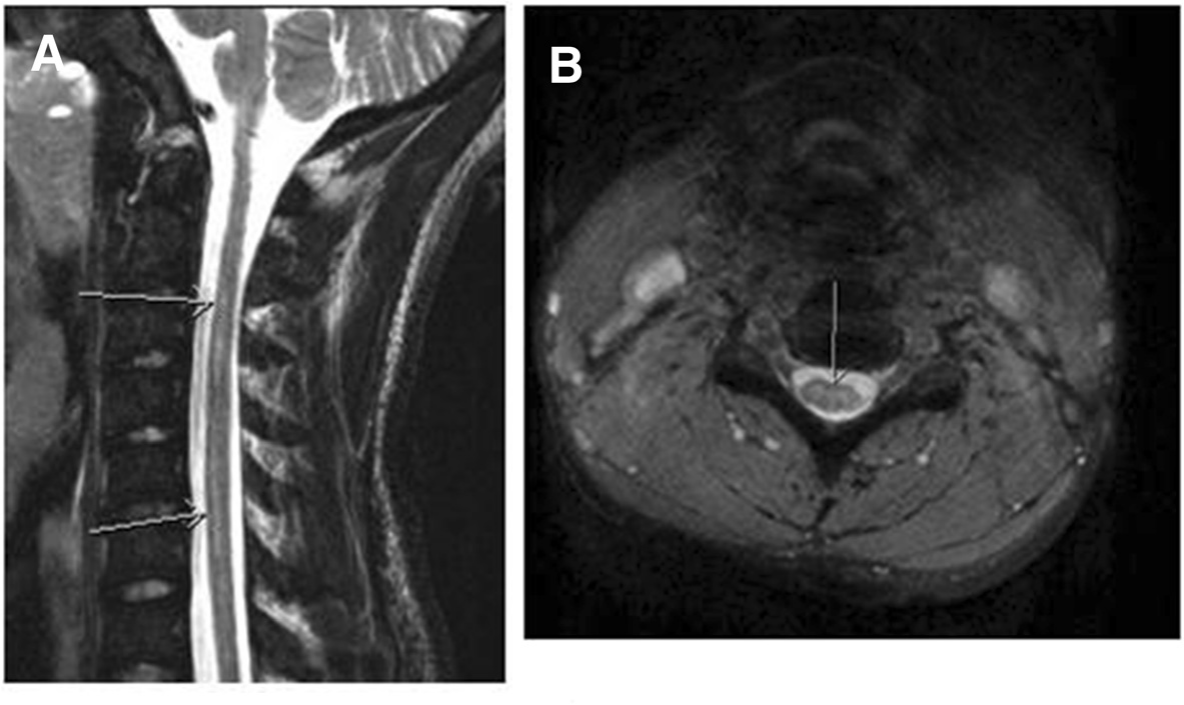
**A,B Follow-up magnetic resonance imaging study demonstrating no spinal cord atrophy with residual signal from myelomalacia, months after infarct.** Arrows point to the decrease in edema.

Five months after the acute onset of paralysis, he had some residual left arm and leg weakness and spasticity, but was able to walk unassisted and perform most activities of daily living without assistance. A repeat MRI scan showed a persistence of slight T2 signal abnormality in the cervical cord, consistent with previous spinal cord infarction. There was no spinal cord atrophy (Figure 2). Our patient continued to make progress, regaining much of his strength and function, and was maintained on a regimen of monthly scheduled exchange transfusions.

At 18 months post-infarct he presented with complaints of three hours of generalized weakness, worse in his lower extremities in association with a pain crisis. His symptoms of weakness had largely resolved by the time he arrived at our Emergency Department. On examination he had 4 out of 5 strength in his left lower extremity and 5 out of 5 in right lower extremity, and 3 out of 5 grip strength bilaterally with a slightly unsteady gait; these findings were not substantially different from his post-spinal cord infarction baseline. His hemoglobin S was 51.5 percent at that time. Repeat imaging studies of his brain and spine at that time were unchanged from his prior studies. He was admitted and had an exchange transfusion achieving a post-transfusion HbS of 8.3 percent. He was given daily low-dose (81mg) aspirin. Currently, at 20 months post-spinal cord infarction, his condition is unchanged.

## Discussion

Spinal cord infarct is infrequent compared to cerebral infarction in the general population, and most commonly occurs as a result of a dissecting aortic aneurysm or aortic surgery [7,8]. In persons with sickle hemoglobin, significant spinal cord infarction appears to be an even more rare neurologic complication. To the best of our knowledge, there are only two reported cases of other persons, both now deceased, detailing this pathology [4,5]. Of note, the radiographic findings from our patient have been previously presented in a radiology journal with emphasis on the diffusion-weighted images, but in this report we describe the clinical details and our patient’s subsequent course [6].

There is a case report from 1970 of a 59-year-old Jamaican woman with presumed sickle cell trait who deteriorated over the course of several years to near complete paraplegia and who was subsequently found to have a slightly swollen spinal cord in the cervical region and atrophic thoracic and lumbar spine cord segments on autopsy [4]. The authors noted that her vasculature and neural tissue was otherwise without the stigmata of significant atherosclerotic or degenerative disease, and while no thrombosed vessels were found in relation to the areas of necrosis in her spinal cord, there were however, many arteries and veins distended with abnormally shaped sickle red cells [4]. A 1980 case report describes a 19-year-old African-American man with sickle cell disease who developed sudden-onset quadriplegia and in post-mortem studies was found to have multiple, old, focal and confluent infarcts involving the cortex and subcortical white matter in the brain, and also of the cervical, thoracic, and upper lumbar spinal cord [5]. There are no data from these case reports in the literature concerning other potential risk factors including any thrombophilic abnormalities, as these were not commonly looked for in 1970 and 1980.

From the available reports that have looked for an association between factor V Leiden and complications of sickle cell disease, there is no evidence of an obvious relationship [16,21,22]. Kahn *et al.* studied a cohort of 82 patients with different sickle cell states, 19 of whom had had a stroke [21]. Only one of the 82 was heterozygous for factor V Leiden (there were no homozygotes), and this was not a patient who had experienced a stroke, priapism or any other vascular-type disorder [21]. Andrade *et al.* similarly examined a cohort of 73 patients with sickle cell disease in Brazil, of whom five had a stroke [16]. One of the five was a heterozygote for factor V Leiden; of the patients who had not experienced a stroke, none were positive for the factor V Leiden mutation. Interestingly, that patient had a sister who also had sickle cell anemia and stroke, but the sister did not carry the factor V Leiden mutation. We conclude that our patient’s heterozygosity for factor V Leiden did not contribute to the occurrence of the spinal cord infarction.

Our patient has severe sickle cell disease as manifested by multiple bouts of recurrent acute chest syndrome and the presence of a silent cerebral infarction. As a comorbidity which predisposes to more severe disease, he also has asthma. However, he would not be considered to be very unusual in having this degree of illness. Therefore, the question arises as to why he developed the rare complication of spinal cord infarction. It occurred during the recovery from an episode of acute chest syndrome, which is known to be a time period of increased risk for cerebral infarction, but this is clearly not a full explanation given the frequency of acute chest syndrome and the rarity of spinal cord infarction. His hypoxemia had resolved when the spinal cord infarction occurred, and his worsened anemia had been corrected. In addition, his sickle hemoglobin percentage was quite low. Although our review of the literature does not suggest that his infarct can be explained by the factor V Leiden heterozygosity, he was not tested for any of the other genetic variants that have been recently found to be associated with stroke in SCD such as ANXA2, TGFBR3, and TEK. It is possible that a combination of factor V Leiden heterozygosity and another mutation may increase his risk for this complication. However, in order to determine risk factors for this complication, its true incidence in SCD must be known.

## Conclusions

It is possible that spinal cord infarction may occur more commonly than previously recognized in sickle cell disease and is missed or misdiagnosed as cerebral infarction. Although in our patient’s case there were clear findings suggestive of spinal cord involvement, some presentations could be more subtle, and many clinicians may not think of the spinal cord when a patient with sickle cell presents with neurologic deficits. We hope that this report may lead others who care for people with sickle cell disease to be vigilant to the possibility of central nervous system infarction involving the spinal cord.

## Consent

Written informed consent was obtained from the patient for publication of this manuscript and any accompanying images. A copy of the written consent is available for review by the Editor-in-Chief of this journal.

## Competing interests

The authors declare they have no competing interests.

## Authors’ contributions

AE reviewed our patient’s case, data and figures, and was a major contributor in writing the manuscript. ELJC reviewed our patient’s case and data, completed subsequent drafts of the manuscript and was a major contributor in writing the manuscript. VJ provided the radiological findings, figures and interpretations. SA was involved during the initial presentation of our patient’s case. RDC was involved during the initial presentation of our patient’s case. RR-L reviewed our patient’s case, data, co-ordinated the authors and was a major contributor in writing the manuscript. All authors read and approved the final manuscript.
